# Draft genome sequence and characterization of *Desulfitobacterium hafniense* PCE-S

**DOI:** 10.1186/1944-3277-10-15

**Published:** 2015-02-24

**Authors:** Tobias Goris, Bastian Hornung, Thomas Kruse, Anika Reinhold, Martin Westermann, Peter J Schaap, Hauke Smidt, Gabriele Diekert

**Affiliations:** 1 Institut für Mikrobiologie, Lehrstuhl für Angewandte und Ökologische Mikrobiologie, University of Jena, Philosophenweg 12, Jena D-07743, Germany; 2 Laboratory of Microbiology, Wageningen University, Wageningen 6703 HB, The Netherlands; 3 Laboratory of Systems and Synthetic Biology, Wageningen University, Wageningen 6703 HB, The Netherlands; 4 Electron Microscopy Center of the University Hospital Jena, Friedrich Schiller University, Jena, Germany

**Keywords:** Anaerobic respiration, Organohalide respiration, Reductive dechlorination, Chlorinated ethenes, Chlorinated phenols, Bioremediation, Reductive dehalogenase

## Abstract

This genome report describes the draft genome and the physiological characteristics of *Desulfitobacterium hafniense* PCE-S, a Gram-positive bacterium known to dechlorinate tetrachloroethene (PCE) to dichloroethene by a PCE reductive dehalogenase. The draft genome has a size of 5,666,696 bp with a G + C content of 47.3%. The genome is very similar to the already sequenced *Desulfitobacterium hafniense* Y51 and the type strain DCB-2. We identified two complete reductive dehalogenase (*rdh*) genes in the genome of *D. hafniense* PCE-S, one of which encodes PceA, the PCE reductive dehalogenase, and is located on a transposon. Interestingly, this transposon structure differs from the PceA-containing transposon of *D. hafniense* Y51. The second *rdh* encodes an unknown reductive dehalogenase, highly similar to *rdhA* 7 found in *D. hafniense* DCB-2, in which the corresponding gene is disrupted. This reductive dehalogenase might be responsible for the reductive dechlorination of 2,4,5-trichlorophenol and pentachlorophenol, which is mediated by *D. hafniense* PCE-S in addition to the reductive dechlorination of PCE.

## Introduction

*Desulfitobacterium* spp. are anaerobic Gram-positive bacteria belonging to the phylum *Firmicutes*. Desulfitobacteria are metabolically versatile bacteria capable of utilizing a wide range of electron donors and acceptors, the latter also including organohalides. Previously, the genome sequences of *Desulfitobacterium hafniense* Y51 and DCB-2 have been published [1,2], and further genomes of various desulfitobacteria are expected to be published in the near future as the result of ongoing sequencing projects (Kruse et al, unpublished results). The genomes of *Desulfitobacterium hafniense* DCB-2 and Y51 are relatively large (5.3 and 5.7 Mbp, respectively) and are characterized by a high number of genes related to energy metabolism. In both genomes, at least one gene encoding a reductive dehalogenase was found. *D. hafniense* DCB-2 contains seven *rdh* genes, two of which are likely non-functional due to either a transposase insertion or a frameshift mutation. The *D. hafniense* Y51 genome harbours one reductive dehalogenase gene, encoding a PCE reductive dehalogenase [1]. Despite the great interest in the potential application of *Desulfitobacterium* spp. and other organohalide-respiring bacteria for bioremediation, only a few reductive dehalogenases have been biochemically characterized. One example of a well-studied reductive dehalogenase is the tetrachloroethene reductase, PceA, from *D. hafniense* PCE-S [3-6].

Here, we describe the isolation and characterization of *D. hafniense* PCE-S together with its draft genome sequence. The organism is capable of dechlorinating PCE via TCE to cis-DCE as well as of several chlorophenols. The draft genome is 5,666,696 bp in size and is compared to the genome sequences of *D. hafniense* Y51 and DCB-2. In addition, some morphological and physiological characteristics of strain PCE-S are given and compared to those of other members of the *Desulfitobacterium* genus.

## Organism information

### Characterization and features

**Figure 1:**
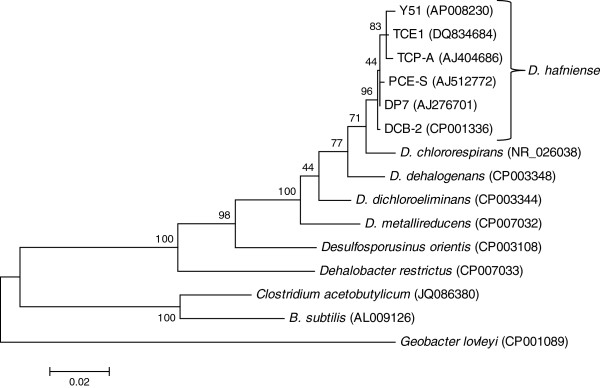
**Phylogenetic tree of *Desulfitobacterium *spp. 16S rRNA gene sequences were derived from NCBI Genbank.** The following 16S rRNA genes were chosen if a complete genome sequence was available: *Bacillus subtilis*: BSU_rRNA_4, *Geobacter lovleyi*: Glov_R0005, *Desulfosporosinus orientis*: Desor_0097, *D. dehalogenans*: Desde_0132; *D. dichloroeliminans*: Desdi_0096, *D. metallireducens*: DESME_00460; *D. hafniense* Y51: DSY_16SrRNA1; *D. hafniense* DCB-2: Dhaf_R0006; *Dehalobacter restrictus*: DEHRE_01135. *D. hafniense* PCE-S 16S rRNA gene sequence: was corrected with the help of the draft genome sequence. The tree was generated with MEGA 6.0 [7] using the maximum likelihood algorithm with 500 bootstraps and standard settings. Sequences were trimmed to the size of the shortest available sequence and aligned with Muscle. Bootstrap values of lower than 70% are considered as low and thus the two nodes with low bootstrap values in the *Desulfitobacterium* clade have to be considered with care.

*Desulfitobacterium hafniense* (Figure 1) PCE-S was isolated from a fixed-bed reactor inoculated with a methanogenic mixed culture, enriched from soil of a dumping site contaminated with chlorinated ethenes. For further enrichment, the mixed culture was immobilized in a fixed-bed reactor with anoxic mineral medium supplemented with 20 mmol l^-1^ ethanol and 0.4 to 0.5 mmol l^-1^ PCE. A pure culture was obtained by inoculating agar medium in roll tubes with a diluted suspension of the biofilm. *D. hafniense* PCE-S has been deposited in the German Collection of Microorganisms and Cell Cultures (DSM 14645).

**Figure 2:**
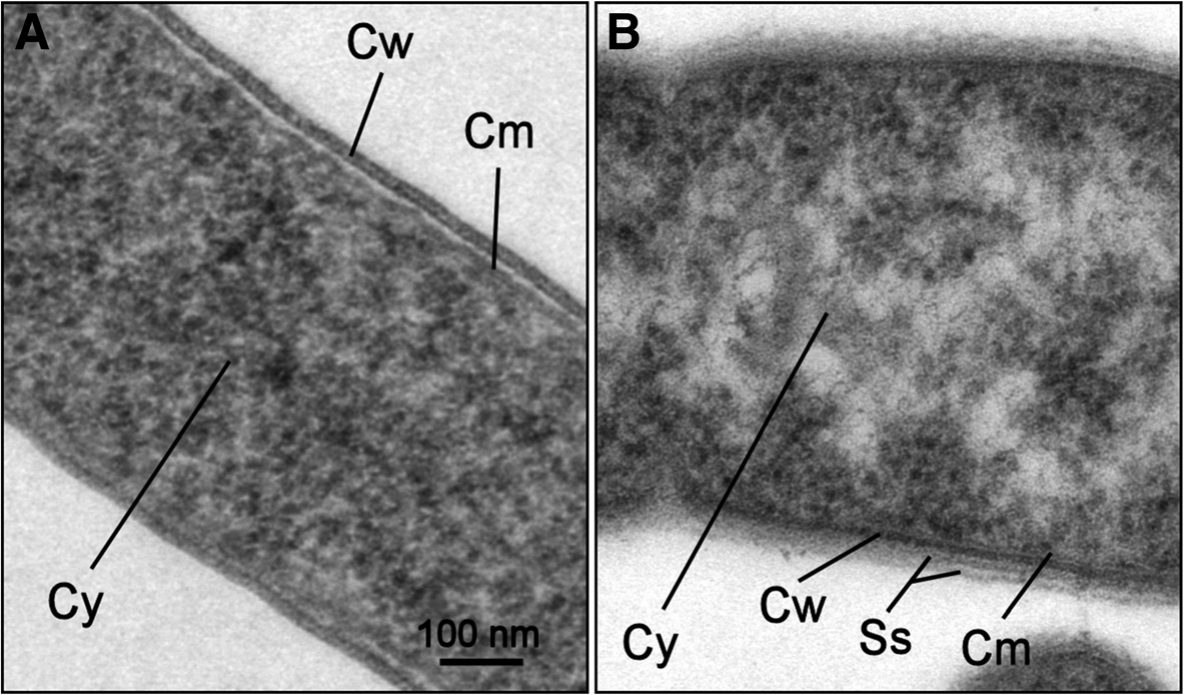
**Ultra-thin section electron micrograph of cells of *D. hafniense *Y51 (A) and PCE-S (B).** Cm: cytoplasmic membrane, Cw: cell wall, Cy: cytoplasm, Ss: slime sacculus (mucosal layer). Cells were grown in the presence of PCE and harvested in the late exponential growth phase. Cells were pre-fixed with 2.5% glutaraldehyde for 1 h and post fixed with 1% osmium tetroxide for 2 h. Samples were dehydrated in ascending ethanol series and embedded in Araldite resin. Ultra-thin sections were prepared with an ultramicrotome (Ultratome III, LKB Produkter AB, Bromma, Sweden) and analysed in a Zeiss EM902A transmission electron microscope (Carl Zeiss AG, Oberkochen, Germany).

*D. hafniense* PCE-S is a slightly curved, sporulating Gram-positive rod of 0.6 μm (diameter) by 6.0 μm (length). Motility was observed only during exponential growth. The cells are surrounded by a slime sacculus, a trait that distinguishes *D. hafniense* PCE-S from *D. hafniense* Y51 (Figure 2). Cytochromes *b* and *c* as well as corrinoids, the latter being an essential cofactor of reductive dehalogenases, were detected in strain PCE-S.

*D. hafniense* PCE-S was shown to utilize pyruvate and several O-methylated compounds (Table 1, [8,9]) as electron donor, whereas acetate, glucose, fructose, mannitol or sorbitol were not utilized as electron donor. Fumarate, nitrate, thiosulfate and several chlorinated compounds were used as electron acceptors (Table 1). In addition, fermentation of pyruvate as sole energy substrate supports growth of *D. hafniense* PCE-S. Growth in liquid media was observed at temperatures ranging from 20°C to 45°C with an optimum at 37°C (Table 1). With pyruvate as electron donor and PCE as electron acceptor, the maximal dechlorination rate was observed at pH 7.7.

**Table 1:** **Classification and general features of *Desulfitobacterium hafniense *** **PCE-S**

**MIGS ID**	**Property**	**Term**	**Evidence code**^**a**^
	Classification	Domain *Bacteria*	TAS [10]
		Phylum *Firmicutes*	TAS [11]
		Class *Clostridia*	TAS [12,13]
		Order *Clostridiales*	TAS [14]
		Family *Peptococcaceae*	TAS [13,15]
		Genus *Desulfitobacterium*	TAS [16]
		Species *Desulfitobacterium hafniense*	TAS [17]
		Strain PCE-S	
	Gram stain	Negative	IDA
	Cell shape	Curved Rods	IDA
	Motility	+ (only exponentially growing cells)	IDA
	Sporulation	+	IDA
	Temperature range	20 – 45°C	IDA
	Optimum temperature	37°C	IDA
	pH range; Optimum	not determined	
	Carbon source	Pyruvate, acetate	IDA
MIGS-6	Habitat	Soil contaminated with chlorinated ethenes	IDA
MIGS-6.3	Salinity	not determined	
MIGS-22	Oxygen requirement	Anaerobic, Microaerotolerant	NAS
MIGS-15	Biotic relationship	free-living	IDA
MIGS-14	Pathogenicity	None known	IDA
MIGS-4	Geographic location	Eppelheim, Germany	IDA
MIGS-5	Sample collection time	1996	IDA
MIGS-4.1	Latitude	49.39	IDA
MIGS-4.2	Longitude	8.62	IDA
MIGS-4.4	Altitude	110 m	IDA

^a)^Evidence codes - IDA: Inferred from Direct Assay; TAS: Traceable Author Statement (i.e., a direct report exists in the literature); NAS: Non-traceable Author Statement (i.e., not directly observed for the living, isolated sample, but based on a generally accepted property for the species, or anecdotal evidence). These evidence codes are from the Gene Ontology project [18].

With PCE as electron acceptor (20 mM, supplied from a hexadecane phase), pyruvate was oxidized to acetate and PCE was dechlorinated to *cis*,1,2-dichloroethene as the main dechlorination product (≥95%) and minor amounts of trichloroethene (≤5%). The chlorinated ethenes were determined gas chromatographically with N_2_ as carrier gas using two bonded-phase fused silica capillary columns.

The generation time of growth with pyruvate as electron donor and PCE as electron acceptor was 10 h without and 8 h with 0.1% yeast extract at 30°C. Fumarate as electron acceptor plus yeast extract led to a slightly shorter generation time (7 h) than with PCE/yeast extract.

The ability of *D. hafniense* PCE-S to dechlorinate polychlorinated phenols was investigated with pyruvate as electron donor and 0.1% yeast extract. Chlorophenols were analysed by HPLC using an RP-18 (5 μm) LiChrospher 100 column (Merck, Darmstadt, Germany). Pentachlorophenol and 2,4,5-trichlorophenol at a concentration of 20 μmol l^-1^ in mineral medium were dechlorinated. 2,4,5-trichlorophenol was partially dechlorinated to 3,4-dichlorophenol, pentachlorophenol was partially dechlorinated to 3,4-dichlorophenol and an unidentified tetrachlorophenol. 2,6-dichlorophenol, 3,5-dichlorophenol, and 2,4-dichlorophenol were not dechlorinated by *D. hafniense* PCE-S.

*D. hafniense* PCE-S has an average nucleotide identity (ANI) of 98.25% to *D. hafniense* Y51 and of 97.6 to the *D. hafniense* type strain DCB-2 [1,2,19].

## Genome sequencing information

### Genome project history

The genome consists of 101 contigs in 24 scaffolds, of which the largest scaffold consists of 5,594,916 bp, covering more than 98% of the genome and more than 98% of the protein coding genes. Table 2 presents the project information and its association with MIGS version 2.0 compliance [20].

**Table 2:** Project information

**MIGS ID**	**Property**	**Term**
MIGS-31	Finishing quality	Improved high quality draft
MIGS-28	Libraries used	One Illumina Miseq paired end library
MIGS-29	Sequencing platforms	Illumina MiSeq Personal Sequencer
MIGS-31.2	Fold coverage	100 ×
MIGS-30	Assemblers	Ray version 2.3, Edena version 3.130110
MIGS-32	Gene calling method	Prodigal version 2.5
	Locus Tag	DPCES
	EMBL ID	LK996017-LK996040
	EMBL Date of Release	September 31, 2014
	GOLD ID	Gp0109025
	BIOPROJECT	264037
	Project relevance	Bioremediation
MIGS 13	Source Material Identifier	DSM 14645

### Growth conditions and DNA preparation

*D. hafniense* PCE-S was cultivated under anoxic conditions as described by Scholz-Muramatsu et al. [21] and Reinhold et al. [22]. For isolation of genomic DNA, *D. hafniense* PCE-S was cultivated for one subculture with fumarate after regularly being cultivated in the presence of PCE. The isolation was carried out as described by Reinhold et al. Approximately 12 μg of genomic DNA were used for genome sequencing. The genome sequence of *Desulfitobacterium hafniense* PCE-S has been deposited in the EMBL database under accession numbers LK996017-LK996040.

### Genome sequencing and annotation

DNA was sequenced at GATC Biotech (Konstanz, Germany) on an Illumina MiSeq Personal Sequencer, generating 1,242,269 paired end reads with a length of 250 bp.

Genome size was estimated prior to assembly using kmer spectrumanalyzer .

The assembly was done in parallel with two different assemblers. One assembly was performed with Edena [23], with standard parameters, the second assembly with Ray, using a kmer-value of 125 [24]. Afterwards both assemblies were merged with Zorro with one of the paired end files supplied [25]. Next, this hybrid assembly was scaffolded with opera version 1.2 [26], which was set up to use Bowtie version 0.12.7 for mapping [27]. As last step, Pilon version 1.4 was used for quality assurance on the assembly [28]. Reads were mapped with Bowtie2 version 2.0.6 [29], further converted with Samtools version 0.1.18 (r982:295) [30] , and then provided to Pilon as input data.

All steps were done using standard parameters, unless stated otherwise. Before annotation, the genome was blasted [31] against itself with an e-value of 0.0001. All contigs with a length of less than 500 bp were discarded, as well as those with less than 1,000 bp which matched onto another genomic location with 100% identity.

After annotation, a check for technical duplications was performed. Contigs, which were determined to be such duplications, were manually removed from the initial assembly and replaced with contigs from the second assembler. The assembly workflow was repeated until no more technical duplications were found.

The assembly was then further scaffolded with CONTIGuator version 2.7.4 [32] and the genome of *Desulfitobacterium hafniense* Y51 as reference [1]. Disagreements with the reference genome were examined with Mauve [33] and Tablet [34], and in case of considerable drops of coverage, the contigs and related reads were isolated, and a re-assembly was performed with Edena. This re-assembly was again scaffolded with CONTIGuator using Y51 as reference genome. Non-scaffolded contigs were included as single contigs in the final result, unless they had a blast hit of more than 90% of their length with a minimum sequence identity of 90% to the scaffold result from CONTIGuator.

The annotation was carried out with an in-house pipeline. In short, this pipeline includes Prodigal version 2.5 for open reading frame identification [35], InterproScan version 5RC7 for protein annotation [36], tRNAscan SE 1.3.1 for tRNA identification [37] and rnammer 1.2 for the prediction of rRNAs [38]. Additional protein function predictions were derived via BLAST [39] UniRef50 and [40] Swissprot databases (downloaded August 2013) [41]. After the annotation process, EC numbers were added with PRIAM version March 06, 2013 [42]. COG assignments were created via blastp best bidirectional hit assignments [43].

## Genome properties

**Figure 3:**
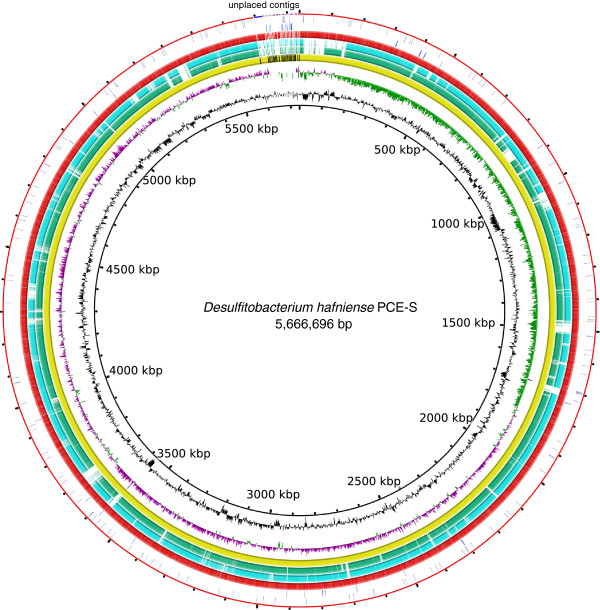
**Circular representation of the genome of *D. hafniense *PCE-S in comparison with strains Y51 and DCB-2.** Rings from inside to outside: (1) GC content (black), (2) GC skew (green/pink), (3) *D. hafniense* PCE-S genome, (3) BLAST comparison with *D. hafniense* Y51 (green) (4) BLAST comparison with *D. hafniense* DCB-2 (turquoise), (5) coding sequences of the genome of *D. hafniense* PCE-S (6) rRNA genes (blue), (7) tRNA genes (violet). The image was generated with BRIG [44].

The genome consists of 24 scaffolds of 5,666,696 bp (47.3% GC content) and an N50 of 5,594,916 bp. In total, 5,494 genes were predicted, 5,417 of which are (Figure 3) protein-coding genes. 4569 of protein coding genes were assigned to a putative function with the remaining annotated as hypothetical proteins. The properties and the statistics of the genome are summarized in Tables 3, 4 and (Additional file 1: Table S1).

**Table 3:** Nucleotide content and gene count levels of the genome

**Attribute**	**Genome (total)**	
	**Value**	**% of total**^**a**^
Genome size (bp)	5,666,696	99.14^b)^
DNA Coding	4,904,707	86.55
DNA G + C (bp)	2,679,309	47.3
DNA scaffolds	24	100
Total genes	5,494	100
Protein-coding genes	5,417	98.47
RNA genes	80	1.42
Pseudogenes	not determined	not determined
Genes in internal clusters	not determined	not determined
Genes with function prediction	4561	83.02
Genes assigned to COGs	3,210	58.26
Genes with Pfam domains	4387	79.85
Genes with signal peptides	296	5.46
Genes with transmembrane helices	1,624	29.98
CRISPR repeats	143	

^a)^The total is based on either the size of the genome in base pairs or the total number of protein coding genes in the annotated genome.

^b)^Percent value of the draft genome sequence compared to the calculated size of the complete genome sequence.

**Table 4:** Number of genes associated with the 25 general COG functional categories

**Code**	**Value**	**% of total**^**a**^	**Description**
J	164	3.03	Translation
A	0	0	RNA processing and modification
K	279	5.15	Transcription
L	168	3.10	Replication, recombination and repair
B	1	0.02	Chromatin structure and dynamics
D	37	0.68	Cell cycle control, mitosis and meiosis
Y	0	0	Nuclear structure
V	81	1.50	Defense mechanisms
T	169	3.12	Signal transduction mechanisms
M	127	2.34	Cell wall/membrane biogenesis
N	57	1.05	Cell motility
Z	1	0.02	Cytoskeleton
W	0	0	Extracellular structures
U	47	0.87	Intracellular trafficking and secretion
O	85	1.57	Posttranslational modification, protein turnover, chaperones
C	237	4.38	Energy production and conversion
G	127	2.34	Carbohydrate transport and metabolism
E	312	5.76	Amino acid transport and metabolism
F	66	1.22	Nucleotide transport and metabolism
H	139	2.57	Coenzyme transport and metabolism
I	77	1.42	Lipid transport and metabolism
P	214	3.95	Inorganic ion transport and metabolism
Q	73	1.35	Secondary metabolites biosynthesis, transport and catabolism
R	415	7.66	General function prediction only
S	280	5.17	Function unknown
-	2,261	41.74	Not in COGs

^a)^The total is based on the total number of protein coding genes in the annotated genome.

## Insights from the genome sequence

Orthologs to other *Desulfitobacterium* species were determined via bidirectional BLAST hits [43] with at least 70% sequence identity and similar size of both sequences (+/- 5%).

**Figure 4:**
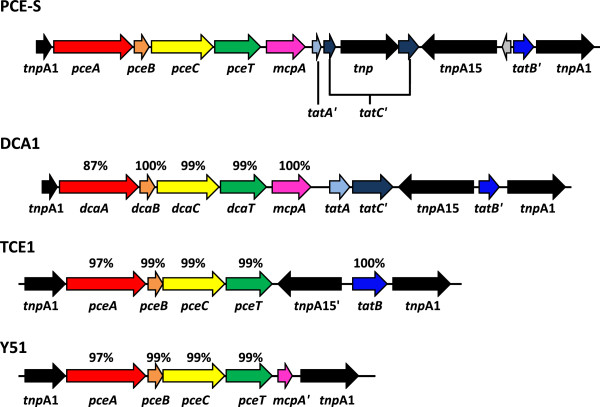
**Comparison of transposons carrying *pceA *or *dcaA *from different *Desulfitobacterium *species and strains**. Values given above the CDS arrows are percentage amino acid identity to the respective PCE-S homologs. Modified and updated after [45].

Two reductive dehalogenase genes (DPCES_1664 and DPCES_3087) are encoded on the genome of *D. hafniense* PCE-S. The latter is the characterized PCE reductive dehalogenase PceA [3]. It is 97% identical (amino acid sequence) to PceA (DSY_2839) from *D. hafniense* Y51, which is located on a transposon. This transposon structure is also found in *D. hafniense* TCE1, where it has been shown to be rapidly lost when the organism is grown in the absence of PCE, leading to the loss of the ability to dechlorinate PCE [45]. The transposon containing *pceA* of *D. hafniense* PCE-S shows a different structure than the one of *D. hafniense* Y51 and TCE1 (Figure 4).

Despite the different organization of this transposon, *D. hafniense* PCE-S also loses the ability to dechlorinate PCE after prolonged cultivation in the absence of PCE [15]. The second reductive dehalogenase gene (DPCES_1664) has no ortholog in Y51. A truncated ortholog is encoded in DCB-2 (Dhaf_2620). In *D. hafniense* DCB-2, the corresponding *rdhA* gene is truncated n-terminally (50 amino acids) due to the insertion of a stop codon through a frameshift mutation. It seems likely that the gene product of DPCES_1664 is responsible for the partial dechlorination of pentachlorophenol and 2,4,5-trichlorophenol by *D. hafniense* PCE-S.

Of the 5,417 protein coding sequences found in the genome of *D. hafniense* PCE-S, 4,402 are orthologous to proteins encoded in either Y51 or DCB-2. *D. hafniense* PCE-S harbours six putative phage regions, of which one was classified as a complete prophage, as detected by PHAST [46]. This is opposed to *D. hafniense* DCB-2 or Y51, where four (DCB-2) and three (Y51) prophages were identified by PHAST as incomplete or questionable, but none as complete. The complete prophage found in *D. hafniense* PCE-S shows highest similarities to *Vibrio* phage X29 (NCBI RefSeq accession no. NC_024369). Several enzymes, of which orthologs fulfill a catabolic function, are not encoded in *D. hafniense* Y51 and DCB-2, but found on the genome of *D. hafniense* PCE-S: An ethanolamine ammonia lyase system (PCES_2016-2020), three molybdopterin oxidoreductase gene clusters (DPCES_4294-6, DPCES_4565-7, DPCES_4582-4), together with a molybdopterin import cluster (DPCES_0024-6), and a protein annotated as cellulose synthase (DPCES_2599). A cluster encoding polysaccharide synthesis enzymes (DPCES_3251 to 3245) might be responsible for the biosynthesis of the slime sacculus of PCE-S.

Five CRISPR regions with a length from 958 to 3415 bp and 14 to 51 spacers were identified in the genome of *D. hafniense* PCE-S with CRISPR finder [47]. This is similar to the situation in DCB-2, where five CRISPR regions with a length of 7 to 60 spacers were found, and in Y51, where five CRISPR regions with a length of 12 to 47 spacers were found. The CRISPR regions in all *Desulfitobacterium* spp. genomes are located in close proximity to each other, separated by not more than 30 kb which are to a large extent covered by CRISPR associated (CAS) proteins.

## Conclusions

Taken together, the genome sequence of *Desulfitobacterium hafniense* PCE-S expands our view on these environmentally interesting microorganisms. The genome sequence gives us insight into the putative chlorophenol dechlorinating activity of a reductive dehalogenase not studied before and might aid bioremediation of chlorinated phenols in the future.

## Abbreviations

PCE: Perchloroethylene or tetrachloroethene; TCE: Trichloroethene; DCE: *cis*-1,2-dichloroethene.

## Competing interests

The authors declared that they have no competing interests.

## Authors’ contributions

TG and GD initiated and supervised the study. TG, BH and TK drafted the manuscript and annotated the genome. AR conducted the wetlab work, MW performed electron microscopy. BH and PJS worked on genome sequencing and assembly. TG, BH, TK, HS and GD discussed, analyzed the data and revised the manuscript. All authors read and approved the final manuscript

## Supplementary Material

Additional file 1: Table S1 Associated MIGS Record. Click here for file
