# Supplementary material for: Draft genome sequence and characterization of Desulfitobacterium hafniense PCE-S
Source: Stand Genomic Sci. 2015 Feb 24;10:15. doi: 10.1186/1944-3277-10-15 (PMC4511579; doi:10.1186/1944-3277-10-15)
Supplement: Additional file 1: Table S1 — Associated MIGS Record. [file 1944-3277-10-15-S1.doc]

***Table S1.*** *Associated MIGS record*

| **MIGS-ID** | field name | description |
| --- | --- | --- |
| **MIGS-1** | Submit to INSDC/Trace archives |  |
| **1.1** | PID |  |
| **1.2** | Trace Archive |  |
| **MIGS-2** | MIGS CHECK LIST TYPE |  |
| **MIGS-3** | Project Name |  |
| **MIGS-4** | Geographic Location | Stuttgart, Germany |
| **4.1** | Latitude |  |
| **4.2** | Longitude |  |
| **4.3** | Depth |  |
| **4.4** | Altitude |  |
| **MIGS-5** | Time of Sample collection | 1996 |
| **MIGS-6** | Habitat (EnvO) | Soil contaminated with chlorinated ethenes |
| **6.1** | temperature |  |
| **6.2** | pH |  |
| **6.3** | salinity |  |
| **6.4** | chlorophyll |  |
| **6.5** | conductivity |  |
|
| **6.6** | light intensity |  |
| **6.7** | dissolved organic carbon (DOC) |  |
| **6.8** | current |  |
| **6.9** | atmospheric data |  |
| **6.10** | density |  |
| **6.11** | alkalinity |  |
| **6.12** | dissolved oxygen |  |
| **6.13** | particulate organic carbon (POC) |  |
| **6.14** | phosphate |  |
| **6.15** | nitrate |  |
| **6.16** | sulfates |  |
| **6.17** | sulfides |  |
| **6.18** | primary production |  |
| **MIGS-7** | Subspecific genetic lineage |  |
| **MIGS-9** | Number of replicons | 1 |
| **MIGS-10** | Extrachromosomal elements |  |
| **MIGS-11** | Estimated Size | 5716086 |
| **MIGS-12** | Reference for biomaterial or Genome report | EBI – Bioproject PRJEB6708 |
| **MIGS-13** | Source material identifiers | DSM 14645 |
| **MIGS-14** | Known Pathogenicity | non-pathogenic |
|
| **MIGS-15** | Biotic Relationship | free-living |
| **MIGS-16** | Specific Host |  |
| **MIGS-17** | Host specificity or range (taxid) |  |
| **MIGS-18** | Health status of Host |  |
| **MIGS-19** | Trophic Level |  |
| **MIGS-22** | Relationship to Oxygen | microaerotolerant |
| **MIGS-23** | Isolation and Growth conditions | *optional: reference may be provided if applicable* |
| **MIGS-27** | Nucleic acid preparation | InnuPREP Bacteria DNA kit (Analytik Jena, Jena, Germany) |
| **MIGS-28** | Library construction |  |
| **28.1** | Library size | One Miseq paired end library, 250 bp, 500 bp insert |
| **28.2** | Number of reads | 1,242,269 |
| **28.3** | vector |  |
| **MIGS-29** | Sequencing method | Illumina MiSeq Personal Sequencer |
| **MIGS-30** | Assembly |  |
| **30.1** | Assembly method | Edena v3.130110, Ray v 2.3 |
| **30.2** | estimated error rate |  |
| **30.3** | method of calculation |  |
| **MIGS-31** | Finishing strategy |  |
| **31.1** | Status | **Improved High Quality draft** |
| **31.2** | coverage | 100x |
| **31.3** | contigs | 101 |
| **MIGS-32** | Relevant SOPs |  |
| **MIGS-33** | Relevant e-resources |  |
